# Purification, characterization and probiotic proliferation effect of exopolysaccharides produced by *Lactiplantibacillus plantarum* HDC-01 isolated from sauerkraut

**DOI:** 10.3389/fmicb.2023.1210302

**Published:** 2023-06-27

**Authors:** Liansheng Yu, Guangbin Ye, Xintong Qi, Yi Yang, Bosen Zhou, Yunye Zhang, Renpeng Du, Jingping Ge, Wenxiang Ping

**Affiliations:** ^1^Engineering Research Center of Agricultural Microbiology Technology, Ministry of Education and Heilongjiang Provincial Key Laboratory of Plant Genetic Engineering and Biological Fermentation Engineering for Cold Region and Key Laboratory of Microbiology, College of Heilongjiang Province and School of Life Sciences, Heilongjiang University, Harbin, China; ^2^Hebei Key Laboratory of Agroecological Safety, Hebei University of Environmental Engineering, Qinhuangdao, China; ^3^School of Basic Medical Sciences, Youjiang Medical University for Nationalities, Baise, China

**Keywords:** *Lactobacillus plantarum*, exopolysaccharides, characterization, purification, application

## Abstract

In this study, an exopolysaccharide (EPS)-producing strain of *Lactiplantibacillus plantarum* HDC-01 was isolated from sauerkraut, and the structure, properties and biological activity of the studied EPS were assessed. The molecular weight of the isolated EPS is 2.505 × 10^6^ Da. Fourier transform infrared spectrometry (FT-IR) and nuclear magnetic resonance (NMR) results showed that the EPS was composed of glucose/glucopyranose subunits linked by an *α*-(1 → 6) glycosidic bond and contained an *α*-(1 → 3) branching structure. X-ray diffraction (XRD) analysis revealed the amorphous nature of the EPS. Scanning electron microscopy (SEM) and atomic force microscopy (AFM) showed that the isolated EPS had a smooth and compact surface with several protrusions of varying lengths and irregularly shaped material. Moreover, the studied EPS showed good thermal stability, water holding capacity, and milk coagulation ability and promoted the growth of probiotics. *L. plantarum* EPS may be used as prebiotics in the fields of food and medicine.

## 1. Introduction

Microbial exopolysaccharides (EPSs) are secondary metabolites released to the extracellular environment during the growth and metabolism of microorganisms, mainly including mucus polysaccharides and capsular polysaccharides (Chen and Huang, 2018; Li et al., 2022). Based on their monosaccharide composition, EPSs are classified as homopolysaccharides consisting of one type of monosaccharide and heteropolysaccharides consisting of two or more types of monosaccharides, such as fructose, mannose, arabinose, rhamnose, xylose and glucose (Daba et al., 2021). The wide range of sources of EPSs results in a wide variation in their structural composition and physicochemical properties, and these differences confer different biological functions (Xu et al., 2019b; Yang et al., 2023). Lactic acid bacteria (LAB), as proven fermenting agents, are extensively used in traditional dairy products, pharmaceuticals, biotechnology and fermentation processes (Wang et al., 2018). LAB are “generally regarded as safe (GRAS)” and have bioactive functions to prevent disease and improve human health; hence, there has been a particular focus on LAB-generated EPSs in recent years (Li et al., 2022). LAB EPSs, with a variety of biological functions, have great potential in the fields of beauty care, the production of chemical materials and health, such as improving the immune system, antioxidation, the ability to produce plastic films, preventing intestinal microbial infections and improving the intestinal microbiological environment, as well as being antiulcer (Barcelos et al., 2020; Rana and Upadhyay, 2020; Wang and Bian, 2020). LAB EPSs have a variety of excellent functional characteristics in the food industry, including improving the taste and rheological properties of fermented foods and bread crumbs and improving the softness of baked products (Daba et al., 2021; Korcz and Varga, 2021).

Similarly, LAB have a complex anabolic system, and LAB EPSs come from a wide range of sources, particularly *Lactococcus lactis*, *Leuconostoc mesenteroides*, *Leuconostoc citreum*, *Lactobacillus casei*, *Weissella*, *Lactobacillus brevis*, *Lactiplantibacillus plantarum*, *Lactobacillus rhamnosus* and others, which are capable of synthesizing a variety of EPSs (Feng et al., 2020; Daba et al., 2021; Allawadhi et al., 2022). LAB EPSs therefore exhibit a great deal of structural diversity. Some specific physicochemical properties and structural composition (molecular weight, monosaccharide composition, three-dimensional structure and type of glycosidic bond, etc.) can confer specific biological activities and physiological functions on EPSs (Angelin and Kavitha, 2020; Du et al., 2022b; Wu et al., 2022). Therefore, revealing the structural composition and physicochemical properties of LAB EPSs is beneficial for further understanding the mechanism of EPS synthesis and for the progress and development of the food and pharmaceutical industries.

*L. plantarum* is an important strain for the food industry, but EPSs produced by different sources of *L. plantarum* vary greatly in structural composition and biological activity (Silva et al., 2019). Therefore, characterizing new sources of *L. plantarum* EPSs is of great importance. In this experiment, the strain *L. plantarum* HDC-01 with high EPS production was isolated from sauerkraut, and its physicochemical properties, structure and functional groups were analysed and characterized.

## 2. Materials and methods

### 2.1. Purification and identification of strain

Sauerkraut was chopped (Zhao et al., 2018), mixed with sterile water and incubated at 30°C for 24 h. Sauerkraut juice was diluted to 10^−7^ with deionized water, inoculated on MRS-S medium (sucrose 20 g/L, beef extract 10 g/L, peptone 10 g/L, yeast 5 g/L, Na_2_SO_3_ 0.1 g/L, MgSO_4_ 0.2 g/L, MnSO_4_ 0.05 g/L, ammonium citrate 2 g/L, KH_2_PO_4_ 2 g/L, CH_3_COONa 5 g/L) and incubated at 30°C for 48 h. Single colonies of mucilaginous-secreting material were picked and inoculated on MRS-S solid medium for purification three times. The purified single colonies were picked out and inoculated onto MRS liquid medium (glucose 20 g/L, beef extract 10 g/L, peptone 10 g/L, yeast 5 g/L, Na_2_SO_3_ 0.1 g/L, MgSO_4_ 0.2 g/L, MnSO_4_ 0.05 g/L, ammonium citrate 2 g/L, KH_2_PO_4_ 2 g/L, CH_3_COONa 5 g/L) and incubated at 140 rpm for 24 h at 30°C.

The purified broth was used to extract the genome using the TIANamp bacteria DNA isolation kit, followed by PCR amplification using 16S universal primers. Primer sequences 5′-TACGGTTACCTTGTTACGACTT-3′ and 5′-AGAGTTTGATCMTGGCTCAG-3′ PCR were performed in a 25 μL reaction system containing 22 μL of 1.1 × T3 super PCR mix, 0.5 μL of each primer and 1 μL of genomic DNA for 30 cycles of amplification. PCR products were detected by 1% agarose gel electrophoresis and sent to the company for sequencing (Du et al., 2018). The sequencing results were compared to GenBank 16S rDNA gene sequences using the BLAST website and submitted to the NCBI database (Accession: OK036442.1). MEGA-based neighbour-joining algorithm for constructing phylogenetic trees of *L. plantarum* HDC-01.

### 2.2. EPS isolation and purification

*L. plantarum* HDC-01 was inoculated onto MRS-S liquid medium and incubated at 30°C for 36 h at 120 rpm. The bacterial fermentation broth was centrifuged at 4°C 12,000 r/min for 30 min to remove the bacteria. Then, 80% (w/v) trichloroacetic acid was added to the supernatant and left overnight at 4°C. The mixture was subsequently centrifuged at 12,000 r/min for 30 min to separate the proteins. Three volumes of precooled 95% ethanol were added to the supernatant, and the EPS was precipitated overnight in a refrigerator at 4°C. The crude EPS was obtained by centrifugation at 12,000 r/min for 20 min at 4°C. The crude EPS was dissolved in deionized water and put into a dialysis bag for dialysis at 4°C for 2 days. The EPS was then fractionated on a Sephadex G-100 gel-filtration chromatography column (1.6 cm × 50 cm) and eluted with deionized water at a flow rate of 2 mL/min. The liquid EPS was frozen at −20°C, and the sample was freeze-dried using a vacuum freeze-dried at −80°C for 12 h (SJIA-10 N-80C, Suangjia, Ningbo) to obtain pure EPS. The EPS solution was prepared with a concentration of 1 mg/mL using deionized water. The UV spectrum was measured by a UV–vis spectrometer (UV-2550, Shimadzu, Japan) in the wavelength range of 190 nm-500 nm to detect the purity of EPS. The EPS content was measured by the phenol–sulfuric acid method (Dubois et al., 1956).

### 2.3. Monosaccharide composition analysis

EPS samples (2 mg) were dissolved in anhydrous methanol containing 1 mol/L hydrochloric acid and hydrolysed at 80°C for 16 h, followed by the addition of 2 mol/L TCA and then hydrolysed at 120°C for 1 h, followed by derivatization with 1-methoxy-2-propylpropionate. Glucose, mannose, rhamnose, galactose and galacturonic acid were used as standards to analyse the monosaccharide composition of the EPS samples by high-performance liquid chromatography (HPLC) (LC20A, Shanghai Shizhong) and to compare the monosaccharide composition of the samples according to the peak times of the standards (Zheng et al., 2014).

### 2.4. Molecular mass analysis

The molecular mass of the purified EPS samples was measured by gel permeation chromatography (GPC; 1,515, Waters, United States). Here, 2.0 mg EPS was dissolved in 1 mL 0.1 mol/L NaNO_3_ solution and filtered through a 0.45 μm cellulose filter. The stationary phase consisted of a porous gel, and the mobile phase was a 0.1 mol/L NaNO_3_ solution with a flow rate of 0.5 mL/min (Du et al., 2022b). Detection was performed by a differential multiangle laser light scattering instrument (DAWN EOS, Wyatt, Shanghai, China) with RI and MALS detectors, a column temperature of 45°C, an Ohpak SB-804 HQ analytical column (F6429103, Ohpak, Shanghai, China), and a loading capacity of 100 μL. The number-average molecular weight (Mn) and mass average molar mass (Mw) of the EPS samples were recorded and processed with GPC/SEC (TDAmaxViscotec) online and offline software. Dextran of different molecular weights (2,700, 9,750, 13,050, 36,800, and 135,350 Da) was used as the standard, and a standard curve was drawn according to the elution peak retention time.

### 2.5. Fourier transform infrared spectroscopy analysis

Freeze-dried EPS powder and KBr were mixed at a ratio of 1:100, ground well and pressed into tablets. The analysis was carried out using FT-IR (Nicolet iS10 spectrometer, United States) with 32 scans in the wavenumber range of 400 to 4,000 cm^−1^ with a resolution of 4 cm^−1^ (Du et al., 2022b).

### 2.6. X-ray diffraction analysis

The freeze-dried EPS samples were laid flat in the cuvette, and their crystal structure was analysed using X-ray diffraction (XRD) (D8, Bruker, United States) in the range of 2θ angles (10°–80°) with a scan rate of 2°/min (Du et al., 2022a).

### 2.7. Scanning electron microscopy analysis

The freeze-dried EPS samples were fixed on conductive adhesive and plated with gold, and their surface structure was observed using scanning electron microscopy (SEM) (S-4800, Hitachi, Tokyo, Japan) with an accelerating voltage of 3 kV at different magnifications (Zhao et al., 2022).

### 2.8. Atomic force micrograph analysis

The EPS solution (1 mg/mL, 5 μL) was dropped onto clean mica sheets and dried with N_2_ airflow. The three-dimensional structure and molecular morphology of the EPS samples were subsequently observed using atomic force micrograph (AFM) (Bruker, Germany) (Zhao et al., 2022).

### 2.9. Nuclear magnetic resonance spectroscopy analysis

The purified EPS samples were dissolved in D_2_O to reach a final concentration of 20 mg/mL EPS solution. 1D nuclear magnetic resonance (NMR) (^1^H-NMR, ^13^C-NMR) and 2D NMR (COSY, NOESY, HMBC, HSQC) were measured using a Bruker AVANCE 600 MHz spectrometer (Bruker, Billerica, United States) at room temperature. Analysis was carried out at 25°C using 400 MHz. D_2_O was used as an internal standard. Chemical shifts were measured in ppm (İspirli et al., 2023).

### 2.10. Thermal analysis

The purified EPS samples were placed in an Al_2_O_3_ crucible. Thermogravimetric analysis (TGA), differential scanning calorimetry (DSC) and derivative thermogravimetry (DTG) were performed on the EPS samples using a Maia F3 200 device (Netzsch, Germany). Experiments were performed in nitrogen at a flow rate of 50 mL/min. Heating was performed at a linear heating rate of 10°C/min over a temperature range of 40–800°C (Wang et al., 2020).

### 2.11. Emulsification activity analysis

The emulsifiability of the EPS samples was determined using Kanamarlapudi’s method (Kanamarlapudi and Muddada, 2017). Diesel, gasoline, hexane, kerosene, benzene, soybean oil, and olive oil (2.5 mL) were mixed with 2.5 mL of EPS solution at a concentration of 2 mg/mL, and the emulsion layer of the mixture was measured at 24 h and 48 h after shaking well for 5 min. The emulsification index (EI) was determined following the formula:


EI=height of the emulsion layer/total height×100.

### 2.12. Viscosity analysis

The effect of concentration (20 mg/mL, 40 mg/mL, 60 mg/mL) and pH (4, 6, 8) on the EPS viscosity was measured using a viscometer at three rotational speeds (6 rpm, 60 rpm, 100 rpm) under room temperature conditions.

### 2.13. Water solubility index and water holding capacity analysis

#### 2.13.1. WSI analysis

In this study, 45 mg EPS samples were dissolved in 0.5 mL deionized water and centrifuged at 12000 rpm for 40 min, the supernatant was removed, and the precipitates were freeze-dried. The weight of the sample after freeze-drying was recorded as M1 (mg) (Wang et al., 2010b). The water solubility index (WSI) calculation formula is as follows:


WSI(%)=[(45−M1)/45]×100

#### 2.13.2. WHC analysis

In this study, 45 g of dried EPS was dissolved in 0.5 mL of deionized water and centrifuged at 12000 rpm for 40 min to obtain the precipitate. The surface water of the precipitate was wiped off with filter paper, and the mass was recorded as W1 (mg). The mass of the precipitate after freeze-drying was recorded as W2 (mg) (Das et al., 2014). The water holding capacity (WHC) calculation formula is as follows:


WHC(%)=(W1/W2)×100

### 2.14. Skimmed milk solidification analysis

Skimmed milk solidification analysis was performed using Kim’s method (Kim et al., 2008). Different concentrations of sucrose (6, 12%) were added to 10% skim milk, followed by inoculation of *L. plantarum* HDC-01 into skim milk at 5% (v/w) inoculum. Skimmed milk without sucrose was used as a control group to observe the degree of curdling capacity of skimmed milk at 24 h and 36 h at 30°C.

### 2.15. Probiotic proliferation test

*L. plantarum*, *Bifidobacterium adolescentis*, *L. casei*, and *Streptococcus thermophilus* were inoculated with MRS liquid medium at 2% inoculum at 30°C for 48 h. The activated four strains were then transferred at 2% inoculum to ferment in MRS proliferation medium with EPS samples, inulin and glucose at a concentration of 20 g/L as the sole carbon source. Samples were taken at 6, 12, 18, 24, 36 and 42 h, and the OD_600 nm_ was measured and data processed using Origin software.

### 2.16. Statistical analysis

The experimental results of this trial were performed in three replicate experiments. All data were processed by Origin 2022 software and the OmicShare Tools online platform, and analysis of variance within and between groups was compared using ANOVA LSD multiple comparisons and t test methods (*p* < 0.05).

## 3. Results and discussion

### 3.1. Isolation and identification of strains

**Figure 1:**
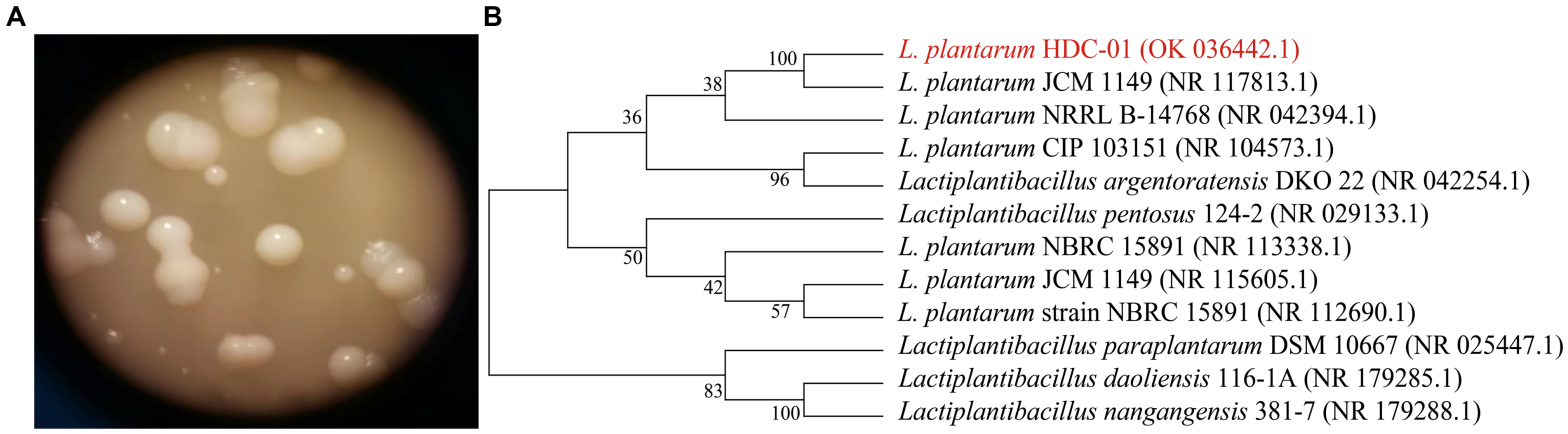
The colony morphology on MRS-S **(A)** and phylogenetic tree of the *Lactiplantibacillus plantarum* HDC-01 **(B)**.

The strain was cultured in MRS-S solid medium and formed a white round, smooth surface, medium-sized colony on the surface of the medium. The presence of sticky material around the colonies indicated that the bacteria produced EPSs (Figure 1A). The colony morphology of the isolated and purified strains conformed to the colony characteristics of LAB. Liu et al. (2019) found that isolated and purified *L. plantarum* HY showed a smooth and hydrated colony appearance on agar plates with a round and medium-sized cell morphology, which was the same as the results of this study.

The 16S rDNA sequences of the bacteria were entered into NCBI for sequence similarity analysis, and the results showed that the sequence information of HDC-01 was 100% similar to that of *L. plantarum* CIP 103151 (Accession: NR_104573.1) and *L. plantarum* 124-2 (Accession: NR_029133.1) with 100% sequence similarity. A phylogenetic tree was constructed for this strain and its close relatives by the NJ method, and the strain was found to have the highest similarity to *L. plantarum* JCM 1149 (Accession: NR117813.1) and *L. plantarum* NRRL B-14768 (Accession: 042394.1) (Figure 1B). In combination with the morphological identification of the above colonies, the strain was identified as *L. plantarum* and named *L. plantarum* HDC-01.

### 3.2. Monosaccharide composition and molecular mass analysis

**Figure 2:**
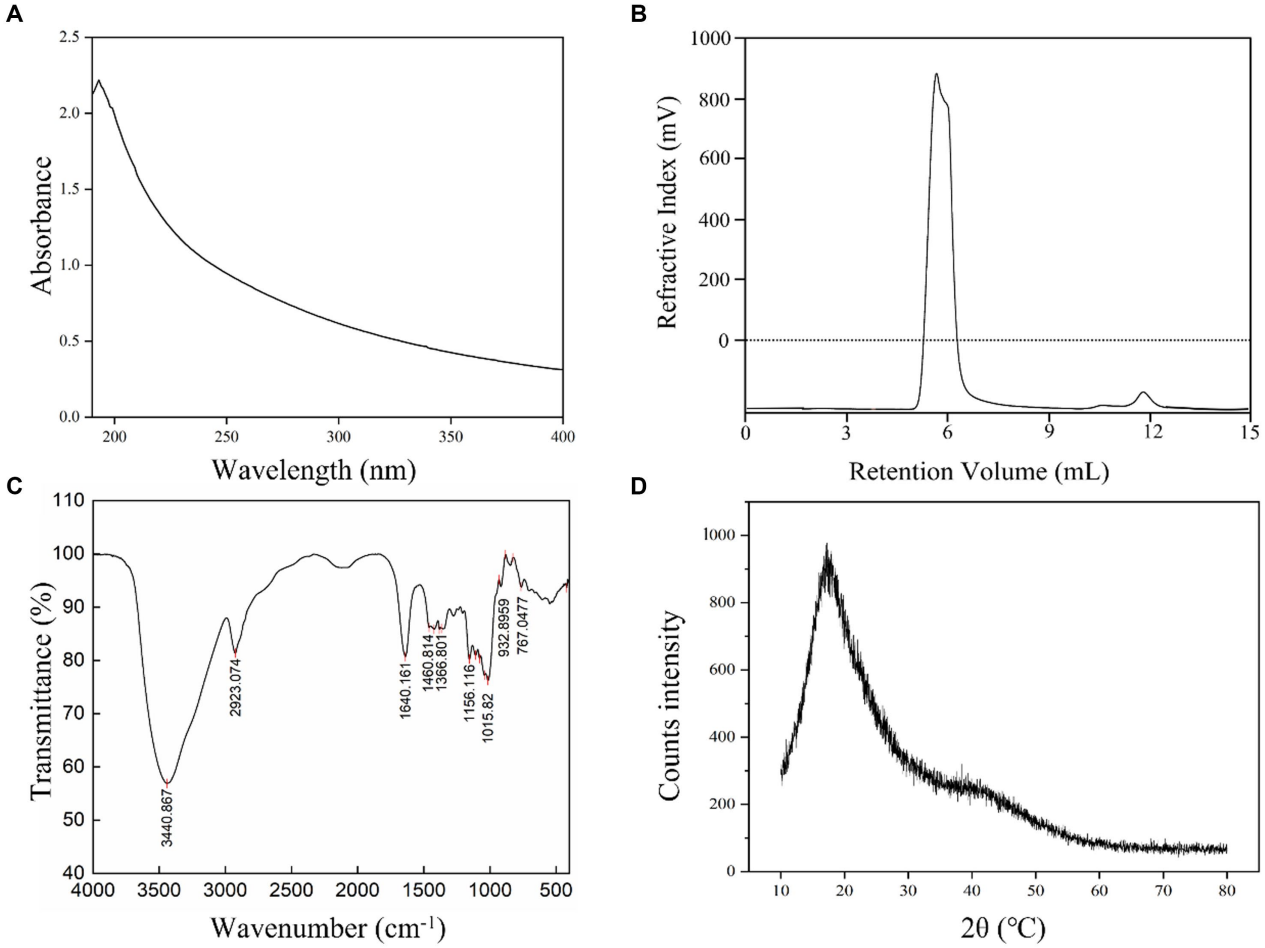
UV **(A)**, GPC **(B)**, FT-IR **(C)** and XRD **(D)** spectrum of the *L. plantarum* HDC-01 EPS.

*L. plantarum* HDC-01 EPS samples were successfully purified by ethanol precipitation, dialysis and a Sephadex G-100 gel-filtration chromatography column. Protein (0%), sulphate and glyoxylate were present in the EPS samples. After the EPS samples were purified, the detector showed only one elution peak for the EPS samples and no absorption peak at 260–280 nm, indicating that the presence of homogeneous EPSs with no nucleic acid or protein contamination (Figure 2A). Freeze-dried EPS appears as a white fluffy solid.

The GPC elution peak showed a single symmetric peak, indicating that the isolated EPS was a homogeneous EPS with high purity (Figure 2B). The Mw and Mn of the EPS were 2.505 × 10^6^ Da and 2.033 × 10^6^ Da, respectively, indicating that the EPS was a low molecular weight EPS, which may improve human immunity and have better solubility and antioxidant properties (Xu et al., 2019b). The Mw of the EPS produced by *L. casei* was 2.7 × 10^6^ Da, similar to the results of this experiment (García-Hernández et al., 2016), but much lower than those produced by *L. plantarum* AR307 (3.85 × 10^6^ Da) and *Weissella confusa* MG1 (7.2 × 10^8^ Da) (Zannini et al., 2013; Feng et al., 2021). Many physicochemical factors, including fermentation conditions, medium composition, substrate concentration, source of polysaccharides, polysaccharide isolation and purification methods, can affect the polymerization of EPSs. Soeiro et al. (2016) found that sucrose as a substrate and inducer of EPS synthesis and different sucrose concentrations significantly affected the Mw of EPSs.

The monosaccharide composition of the isolated EPS showed that the monosaccharide structure was glucose. Combined with the GPC chromatogram, the sample was a homopolysaccharide composed of glucose. *L. brevis* HDE-9 and *W. confuse* H2 produced EPSs composed of glucose, which was the same as the results of this experiment (Du et al., 2022a,b). In addition, EPSs produced by some LAB are heteropolysaccharides composed of different monosaccharide structures. Do et al. (2020) found that EPS-W1 produced by *L. plantarum* was a heteropolysaccharide composed of glucose and mannose. The SSC-12 EPS produced by *Pseudomonas pentosus* was a heteropolysaccharide composed of glucose, mannose, galactose, arabinose, and rhamnose (Fan et al., 2021). These results were different from those of the present experiment.

### 3.3. FT-IR spectroscopy and XRD analysis

The functional group composition of the EPS samples was analysed by FT-IR. The results showed that the EPS samples displayed multiple absorption peaks in the FT-IR spectrum in the range of 400–4,000 cm^−1^ (Figure 2C). A strong absorption peak was identified at 3440.867 cm^−1^, which was caused by the presence of a large number of O–H stretching vibrations in the EPS samples. The strong absorption peak at 2923.074 cm^−1^ was due to the C–H variable angle vibration in the EPS and was a characteristic peak for polysaccharides (Karadeniz et al., 2021). The absorption peak observed at 1640.161 cm^−1^ corresponded to the stretching vibration of COO^−^. The absorption peaks of various types in the fingerprint region at 1200–950 cm^−1^ demonstrated specific structural information of the isolated EPS, the absorption peak at 1015.82 cm^−1^ indicated the presence of *α*-(1 → 6) glycosidic bonds in the EPS, and 932.896 cm^−1^ represented the presence of *α*-(1 → 3) glycosidic bonds. The variable angle vibration at 767.0477 cm^−1^ was the result of the pyranose ring (Ye et al., 2012). Thus, FT-IR analysis showed that the EPS contained characteristic absorption peaks for most of the polysaccharides (Wang et al., 2021).

XRD can reveal information about the phase structure of EPSs and help to understand the physical properties of EPSs. The XRD pattern showed very broad and asymmetrical strong diffraction peaks at 20°C (2θ), indicating many noncrystalline regions in the EPS samples and a small number of crystalline regions within the amorphous regions (Figure 2D). Thus, the XRD spectrum shows the isolated EPS in a noncrystalline amorphous state, and this result was consistent with dextran produced by *Lactobacillus kunkeei* AP-27 and *L. kunkeei* AP-37 (İspirli et al., 2023; Yilmaz et al., 2023).

### 3.4. SEM and AFM analysis

**Figure 3:**
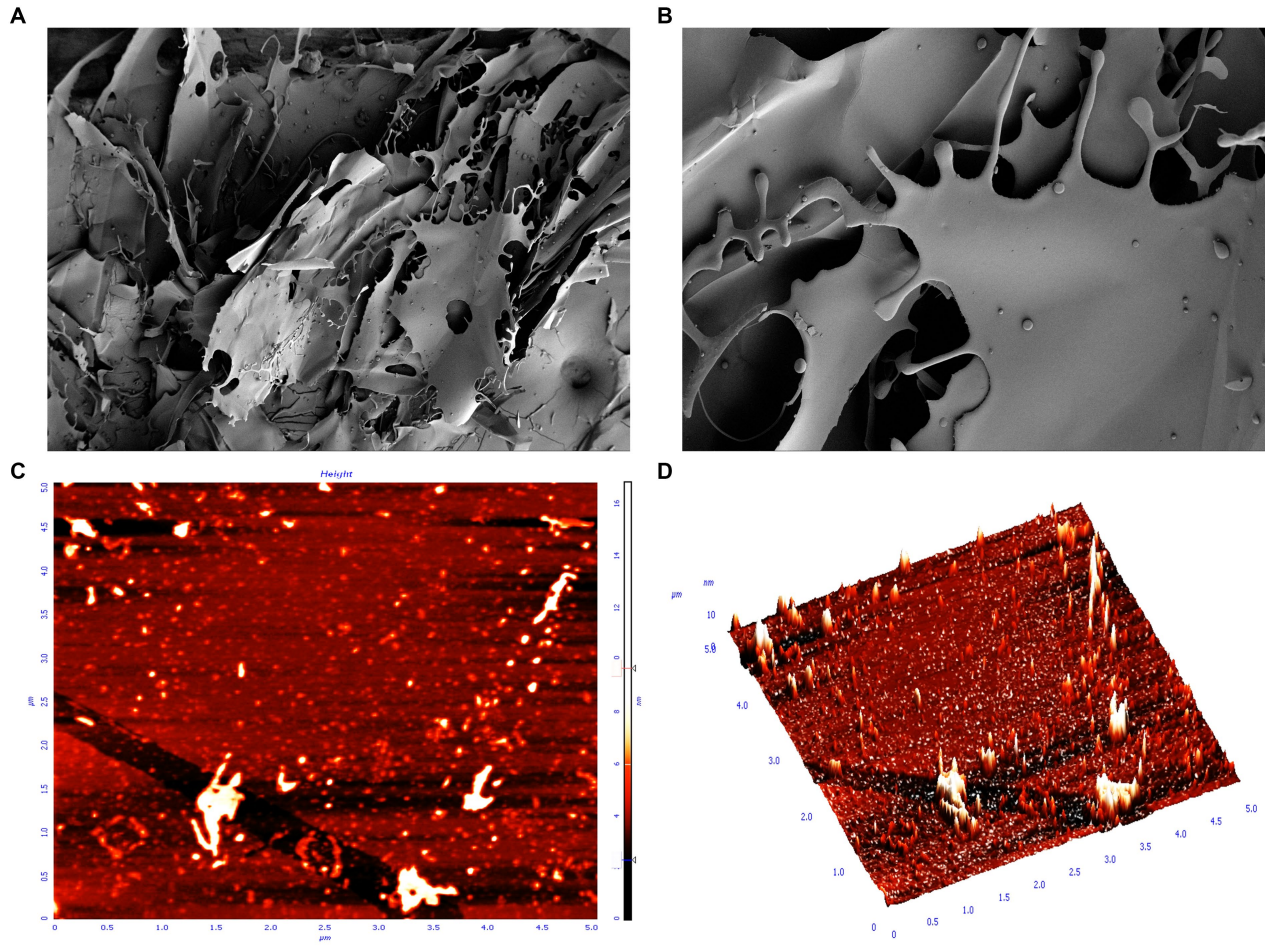
SEM and AFM display of the surface morphology of EPS. **(A)** SEM magnification ×100; **(B)** SEM magnification ×500; **(C)** AFM plan view; **(D)** AFM cubic view.

SEM can help to study the microstructure and surface morphology of EPSs, which helps to understand the physical properties of EPSs (Yu et al., 2022). The SEM results are shown in Figures 3A,B. The EPS structure was an irregular, smooth and continuous mesh-like sheet structure. By using a higher magnification, the EPS surface structure was observed to be flatter and smoother, indicating that EPSs can improve the rheological properties of food and promote viscosity and water holding capacity (Xu et al., 2019b; Yang et al., 2023). The irregular and continuous mesh-like sheet structure gives EPSs good mechanical stability, and the smooth structure gives EPSs the ability to make pliable film materials. Similar EPS structures have been found in *Ln. citreum* B-2, *W. confusa* H2, *Ln. mesenteroides* XR1, and *L. plantarum* KF5 (Wang et al., 2010a; Xu et al., 2020; Wang et al., 2021; Du et al., 2022a). Numerous studies have confirmed that such EPSs can be used in the food field as thickeners and emulsifiers and have the ability to retain moisture in food and produce plastic films (Srinivash et al., 2023).

AFM provides further insight into the morphological features; 3D structure and dynamics of EPSs and is an important tool for studying the structural properties of EPSs (Du et al., 2023). AFM provides EPS images with a scale of 5.0 μm × 5.0 μm and height of 10 nm. The results showed that the isolated EPS had a rough surface, with protrusions of varying lengths and some irregular substances (Figures 3C,D). The rough surface indicated that the EPS samples had good water holding capacity and biocompatibility and could be widely used in the pharmaceutical field. The protrusions of variable length may be certain regions or side chains of polysaccharide molecular chains, which may have specific functions, such as the ability to interact with other molecules and regulate the stability and solubility of polysaccharides (Xu et al., 2019a; Allawadhi et al., 2022). In addition, these protrusions may also affect the morphology and stability of polysaccharides, thus affecting their function and application in living organisms (Xu et al., 2019a). The irregular shape may be a microstructure formed by the self-assembly of polysaccharide molecules or a complex formed by the interaction of polysaccharides with other molecules (e.g., proteins, phospholipids, etc.) (Wang et al., 2023).

### 3.5. Nuclear magnetic resonance spectroscopy analysis

**Figure 4:**
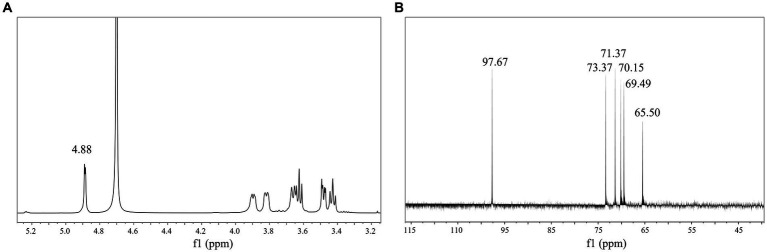
1D NMR spectrum of EPS. **(A)** ^1^H NMR spectrum; **(B)** ^13^C NMR spectrum.

NMR can further reveal the structure and composition of EPSs. Figure 4A shows the ^1^H NMR spectrum of the isolated EPS, and the results indicated that the signal was mainly concentrated at *δ* 3.3–5.0 ppm. The spectrum consisted of an anomalous region (*δ* 4.6–5.0 ppm) and a ring proton region (*δ* 3.3–4.0 ppm). The chemical shifts in the ring proton region were shielded by the hydroxyl group, the signal peaks were poorly separated, and the signal overlap was severe, leading to difficulties in resolution. The signal peak between *δ* 3.3–4.0 ppm was assigned to H_2_-H_6_ on glucose residues. The presence of a strong signal peak at *δ* 4.5–5.5 ppm indicated that the isolated EPS was a homopolysaccharide composed of monosaccharides. A similar structure was found in both dextran produced by *W. confusa* and EPSs produced by *L. brevis* HDE-9 (Du et al., 2022a, 2023). The strong signal peak at *δ* 4.88 ppm indicated that the isolated EPS was a pyranose consisting of *α*-(1 → 6)-glycosidic bonds, and the low signal anomaly peak at *δ* 5.1–5.3 ppm indicated the presence of *α*-(1 → 3)-glycosidic bonds. Moreover, the presence of multiple signal peaks at *δ* 4.5–5.5 ppm implied that the isolated EPS was a heteropolysaccharide consisting of multiple monosaccharides. The EPS produced by *L. plantarum* W1 had six ectopic proton signals (*δ* 5.76, 5.67, 5.59, 5.59, 5.56, 5.55, 5.37 ppm) in the anomalous region (*δ* 4.5–5.5 ppm), showing that the EPS samples were composed of seven monosaccharides of heteropolysaccharides (Do et al., 2020), which was different from the results of this test. Therefore, the isolated EPS was inferred to be a pyranose consisting of *α*-(1 → 6) and *α*-(1 → 3) glycosidic bonds.

The ^13^C NMR results indicated the presence of signals in the anomalous carbon region (*δ* 95–110 ppm) versus the cyclic carbon region (*δ* 50–85 ppm) and corresponded to the anomalous region (*δ* 4.6–5.0 ppm) and the cyclic proton region (*δ* 3.3–4.0 ppm) in the ^1^H NMR spectrum. The signal value at *δ* 97.67 ppm indicated that the isolated EPS was *α*-D-pyranose. The signal values at *δ* 73.37, 71.37, 70.15, 69.49, 65.50 ppm corresponded to glucose residues C_3_, C_2_, C_5_, C_4_, and C_6_ (Figure 4B). The signal value at *δ* 65.50 ppm implied that the EPS samples contained *α*-(1 → 6) glycosidic bonds. The spectra showed no signal peaks at *δ* 107–109 ppm and *δ* 80–85 ppm, indicating that the isolated EPS did not contain furanose. Combined with the FT-IR and ^1^H NMR results, the EPS was a homopolysaccharide linked by an *α*-D-(1 → 6) glycosidic bond and contained an *α*-(1 → 3) branching structure. The EPS produced by *Leuconostoc pseudomesenteroides* contained 97.3% *α*-D-(1 → 6)-linked glucose and had 2.7% *α*-(1 → 3)-branches, and the EPS produced by *Ln. mesenteroides* NTM048 consisted of *α*-(1 → 6)-linked glucose with *α*-(1 → 3)-branches, which was similar to the results of this experiment (Matsuzaki et al., 2017; Du et al., 2023).

**Figure 5:**
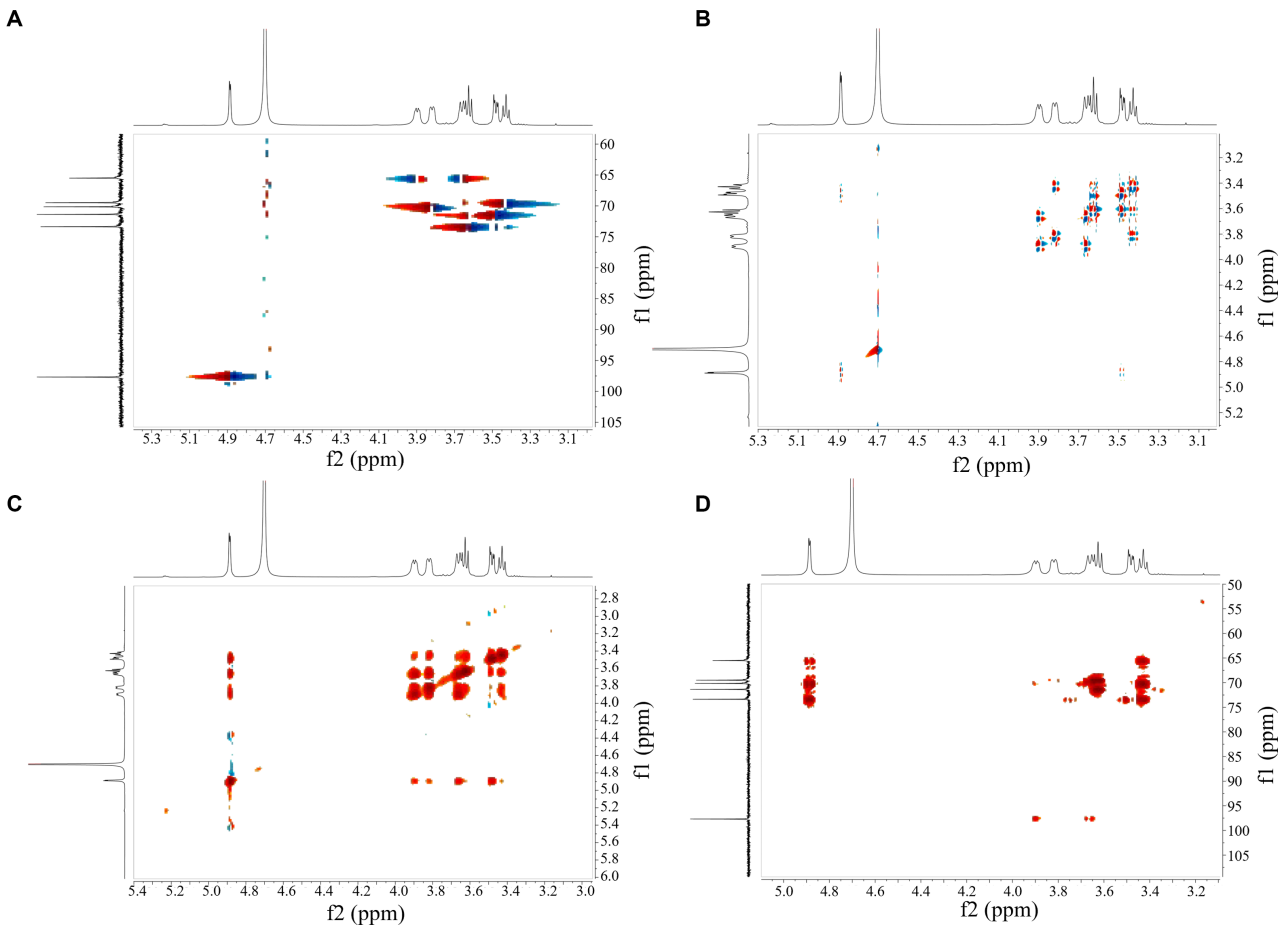
2D NMR spectrum of EPS. **(A)** HSQC, **(B)** COSY, **(C)** NOESY and **(D)** HMBC.

HSQC showed peak signals at 4.87/97.55 (H_1_/C_1_), 3.47/71.46 (H_2_/C_2_), 3.61/73.36 (H_3_/C_3_), 3.41/69.54 (H_4_/C_4_), 3.85/69.68 (H_5_/C_5_), 3.93, and 3.65/65.43 (H_6_, H_6_/C_6_) (Figure 5A). Combining COSY and HSQC maps revealed the chemical shifts of C_2_–C_6_ and H_2_-H_6_ of EPS, confirming the presence of *α*-(1 → 6) residues and *α*-(1 → 3) branch structures in the repeating units of EPS (Figure 5B). COSY profiles calculated ^1^H–^1^H coupling constant values for JH_2_, H_3_, JH_3_, H_4_ and JH_4_, JH_5,_ respectively, confirming that the isolated EPS was a glucose in the form of pyranose. Inter-residue correlations were obtained from NOESY (Figure 5C) and HMBC (Figure 5D) spectra, validating the results of the COSY and HSQC spectra.

### 3.6. Thermal analysis

**Figure 6:**
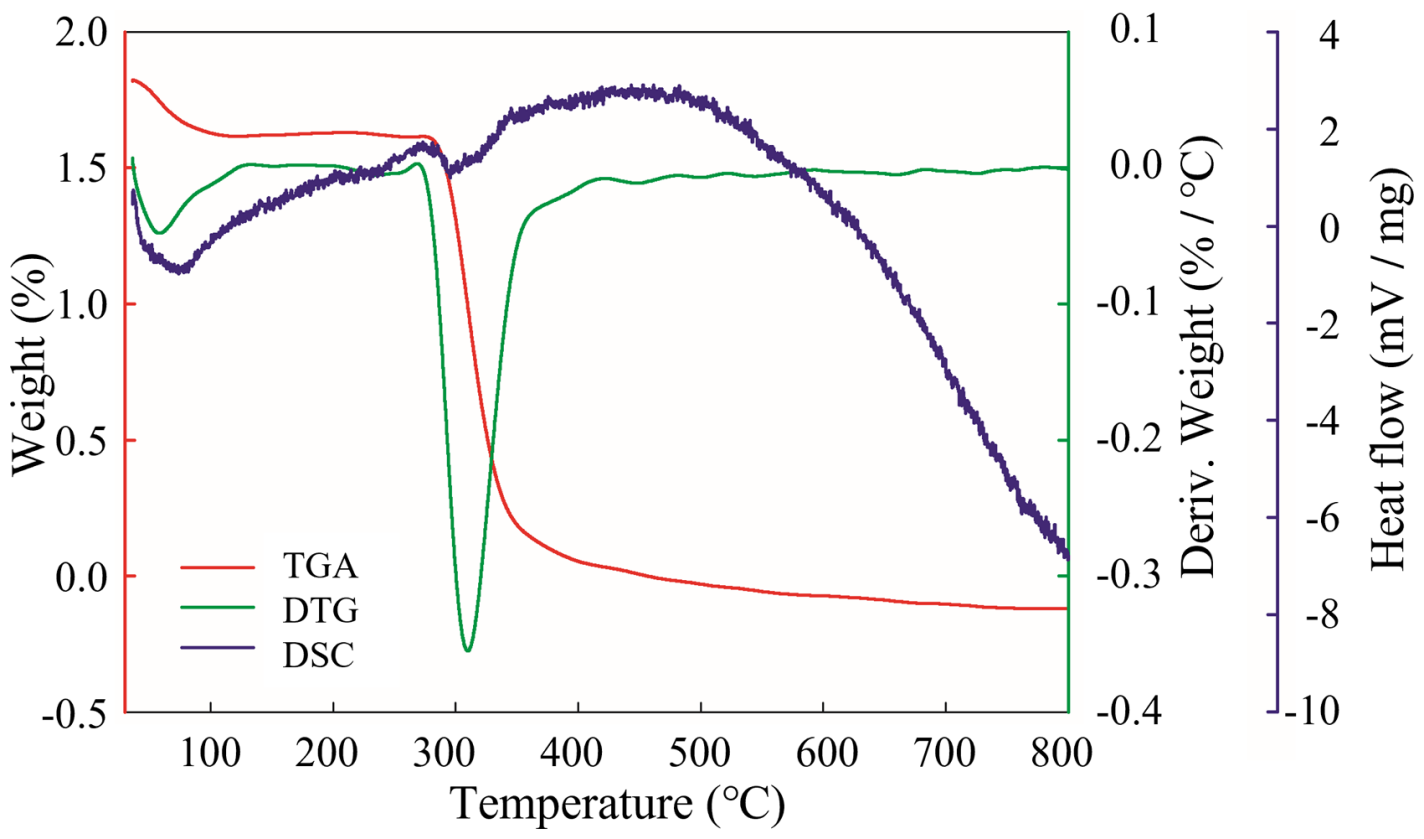
EPS thermal performance analysis. Red curve is TGA; green curve is DTG; purple curve is DSC.

The analysis of the thermal properties will help to further reveal the physicochemical properties of EPSs and expand the application of EPSs in industry. The TGA results are shown in Figure 6, and EPS degradation was divided into three main stages. First, a weight penalty of approximately 10% was observed between 30°C–100°C. This was because the isolated EPS contained many carboxyl groups, and the increase in temperature led to a loss of bound water, which reduced the weight of the EPS (Du et al., 2022b). Second, at 300°C–400°C, the EPS suffered a weight penalty of 41.41%. This was caused by the effect of high temperatures on the EPS and thus depolymerization, breaking of the C–C and C=O bonds in the ring unit, production of water molecules and volatilization, resulting in a dramatic reduction in weight (Du et al., 2022a). The weight of the EPS was maintained at a stable level at 400°C–700°C. The DTG curve indicated a degradation temperature (Td) of 309.86°C for the isolated EPS, which was higher than *Ln. mesenteroides* DRP-5 dextran (298.1°C) (Du et al., 2018), *L. plantarum* CNPC003 EPS (210°C) (Bomfim et al., 2020) and *W. confusa* PP29 dextran (305°C) (Rosca et al., 2018). As the temperature increased further, the EPS weight of remained relatively constant. This phenomenon was related to the complexity of the EPS molecular structure, monosaccharide composition and molecular weight. The higher degradation temperature indicated that the isolated EPS was thermally stable and has great potential for use in the food chemical industry. The DSC curve showed a clear endothermic peak of melting at 100.22°C during the initial phase of heating, which was associated with the evaporation of water and the melting of the crystalline structure formed by the long aliphatic side chains of the EPS molecule (Bomfim et al., 2020). The DSC curve was consistent with the TGA and DTG curves, indicating that EPSs can be used in a wide range of applications in the food, pharmaceutical and chemical industries.

### 3.7. EA analysis

Emulsifiers can maintain the stability of two immiscible liquid phase systems and are therefore widely used in food and chemical industries. As shown in Table 1, the overall emulsification trend was gasoline> soybean oil> kerosene> benzene> hexane> olive oil> diesel. There were significant differences in the emulsifying activity of different hydrocarbons and oils. Among them, gasoline had the strongest emulsifying activity and showed very good dispersion properties, while olive oil had the weakest emulsifying activity. This may be because gasoline contains many low molecular weight compounds that were better able to interact with the EPS and thus enhance the emulsifying activity. Olive oil was less dispersed due to its high molecular weight and low surface activity. Moreover, the emulsifying activity of all tested samples was higher after 48 h than at 24 h. This may be because hydrocarbons and oils were able to interact better with the EPS over a longer period of time, thus enhancing the emulsification activity (EA). The emulsifying properties of the isolated EPS were similar to the results of *Leuconostoc pseudointestinalis* HDL-3, *L. brevis* HDE-9, and *W. confusa* H2 (Zhao et al., 2022; Du et al., 2022a,b).

**Table 1:** Emulsification activity (EA) of exopolysaccharide (EPS) with hydrocarbons and oils E24, 24 h; E48, 48 h.

Hydrocarbons/oil	EA (%)	
	24 h	48 h
Diesel	52.30 ± 1.82	56.92 ± 1.02
Gasoline	73.67 ± 1.09	84.90 ± 2.64
Hexane	60.73 ± 2.98	75.73 ± 2.45
Kerosene	64.09 ± 1.76	77.71 ± 1.37
Benzene	60.01 ± 2.87	76.23 ± 2.61
Soybean oil	80.34 ± 2.62	82.98 ± 2.08
Olive oil	57.83 ± 1.29	62.93 ± 1.06

### 3.8. EPS rheological properties

**Figure 7:**
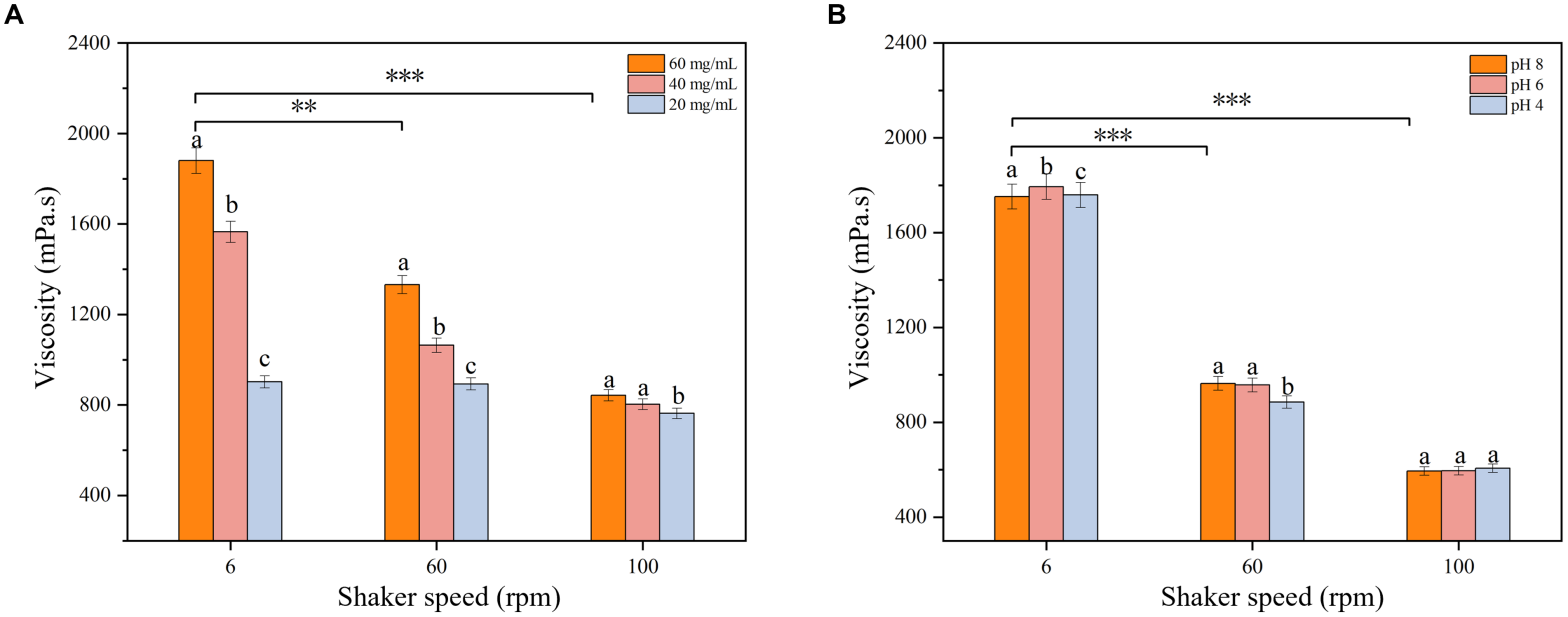
Effect of **(A)** concentration and **(B)** pH on EPS viscosity. The represent a-c the significant difference of different pH and concentration values on EPS viscosity at the same Rev. The star represents the significant difference of EPS concentration and pH on EPS viscosity at different speed (*p* < 0.05).

Rheological property analysis can help to study the biological function of EPSs and provide a theoretical basis for the industrial application of EPSs. The viscosity analysis of the isolated EPS is shown in Figure 6. The viscosity decreased gradually with increasing rotational speed, showing the shear dilution characteristics of non-Newtonian fluids (Du et al., 2022a). When the speed of the shaker increased, the interaction force between the EPS molecules weakened, and the molecular spacing increased, which led to a reduction in frictional resistance and adhesion between the EPS molecules and a decrease in viscosity. The EPS viscosity was measured at different EPS concentrations and pH values, and the results showed that the viscosity was maximum at an EPS concentration of 60 mg/mL (Figure 7A). The high EPS concentration increased the number of crosslinks between the molecular chains and the frictional resistance and adhesion between the crosslinking points, further enhancing the EPS viscosity. The EPS viscosity at a pH of 6 was higher than at pH values of 4 and 8 (Figure 7B), which was due to the lower ionization of the EPS at a pH of 6. The charge state of the carboxyl and amino groups on the molecular chains was in the most balanced state, when the electrostatic force on the molecular chains was minimal and the interaction between the molecular chains was strongest, which led to the highest EPS viscosity. When the pH value was higher or lower than 6, most of the carboxyl groups in the isolated EPS lost their negative charge or most of the amino groups lost their positive charge, and the interaction force between the molecular chains was weakened, resulting in a decrease in viscosity. Moreover, changes in pH also affect the conformation of EPSs. When the pH changes, the interactions of hydrogen bonds, ionic bonds and van der Waals forces on the EPS molecule also change, resulting in a change in the conformation of the molecular chains, which also affects the EPS viscosity (Wu et al., 2023).

### 3.9. WSI and WHC analysis

The WSI and WHC of the EPS samples were 98.73 ± 2.63% and 464.76 ± 9.03%, respectively, which were higher than those of *Ln. pseudomesenteroides* and *Ln. lactis* KC1174 (Saravanan and Shetty, 2016; Du et al., 2022a). The higher WSI and WHC were because the EPS samples contained a large number of glucose units of hydroxyl groups, which could bond with hydrogen to form a large amount of water. The EPS showed good hydrophilicity and water retention ability, so it could improve the texture and rheological properties of food or be applied as a biosurfactant and stabilizer in the chemical industry and other fields.

### 3.10. Skimmed milk solidification analysis

**Figure 8:**
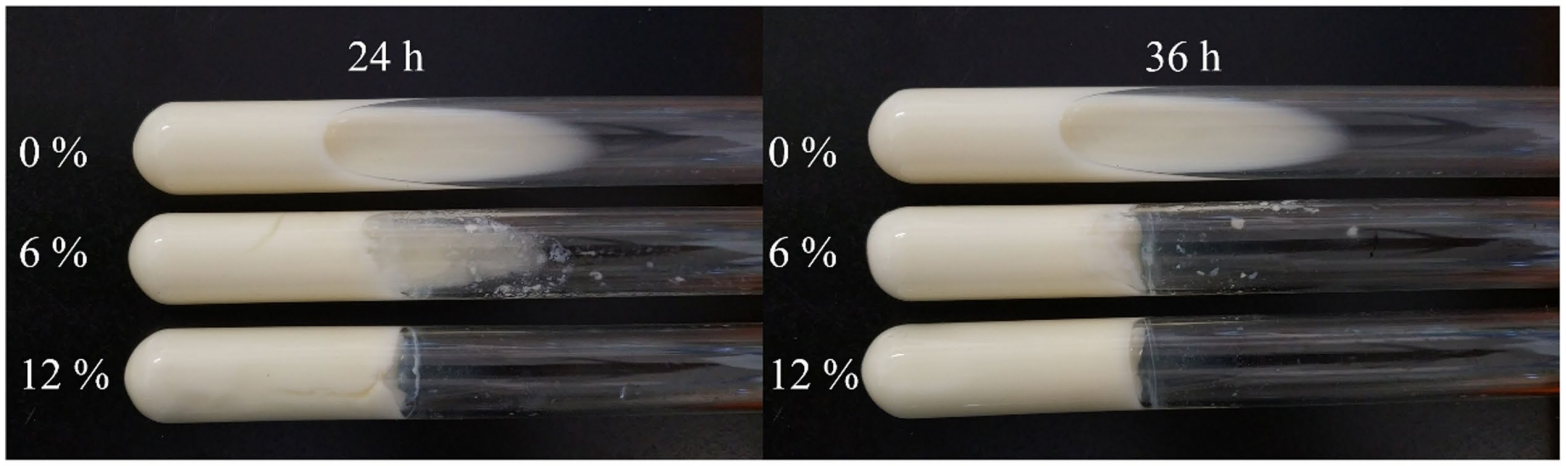
Degree of milk solidification of strains at different sucrose concentrations.

The results of the skimmed milk solidification test are shown in Figure 8, with no solidification occurring in the control group at 12 and 24 h. The degree of milk solidification in the experimental group was enhanced with increasing sucrose concentration and longer fermentation time, indicating that the strain could produce EPSs through sucrose and promote the solidification of skimmed milk. The degree of milk solidification depended on the initial sucrose concentration and the fermentation time. This result was similar to those for *L. brevis* HDE-9, *Lactiplantibacillus paraplantarum* NCCP 962 and *Lactiplantibacillus pentosus* B8 (Jiang et al., 2022; Du et al., 2022b; Afreen et al., 2023). The milk solidification was because the EPS produced by the strain interacted with the proteins in skimmed milk, causing changes in the spatial structure of the proteins. The level of milk solidification was related to the physicochemical properties of the EPS, the kind of protein in the skimmed milk and the ratio of EPS to milk. Wang et al. found that the highest yield of *Ln. citreum* B-2 EPS and the best solidification of skimmed milk was achieved when the sucrose content was 9% (w/v) (Wang et al., 2020). The strains can be used as fermenters and food additives for dairy products, thus improving the taste as the rheological properties of food products. Experiments with skim milk solidification reveal the industrial potential of EPSs.

### 3.11. Probiotic proliferation

*L. plantarum*, *L. casei*, *S. thermophilus*, and *B. adolescentis* are the most common probiotics in the human intestine, ensuring intestinal homeostasis and maintaining normal intestinal barrier function. They have a good tolerance to acid and bile and can fight gastrointestinal diseases. Moreover, the ability of probiotics to adhere to the intestinal mucosa and/or extracellular matrix components helps to inhibit pathogen colonization and improve mucosal healing. The growth of probiotics is usually proliferated by LAB EPSs and functions as a cofactor to help the probiotic flora maintain a healthy gut.

**Figure 9:**
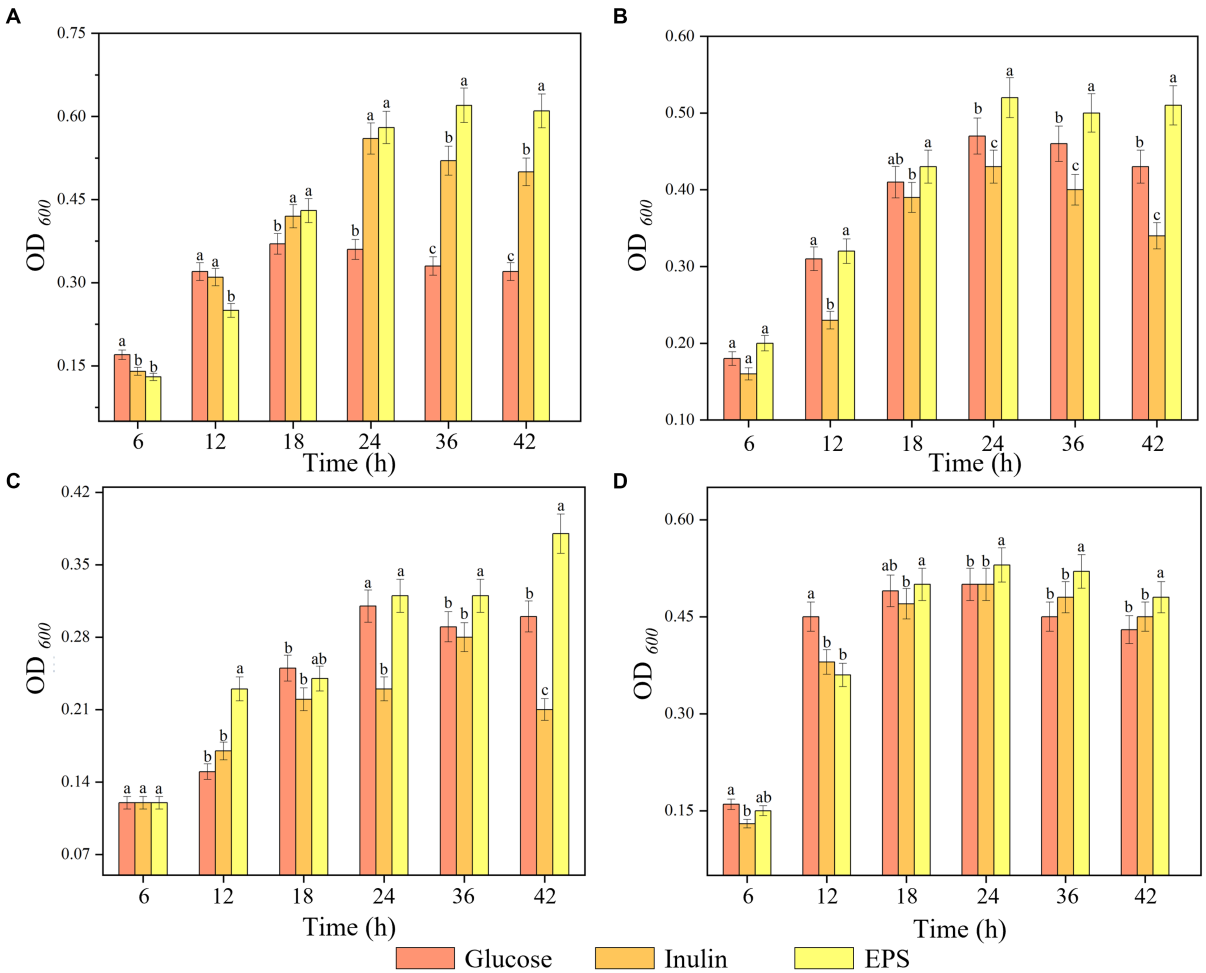
Proliferation of strains by three substrates. **(A)** *L. plantarum*, **(B)** *L. casei*, **(C)** *S. thermophilus*, **(D)** *B. adolescentic.* a-c Indicates the proliferation capacity of different polysaccharides for probiotics at the same time.

The proliferative effect of three carbohydrates on four probiotics was tested, and the results are shown in Figure 9. The proliferation of the four strains by the different carbohydrates differed significantly (*p* < 0.05), among which the EPS significantly promoted the proliferation of probiotics. Three carbohydrates showed no significant proliferative effect on probiotics in the first 6 h, but probiotics grew rapidly in the 24–36 h. Compared with inulin and glucose, the isolated EPS could significantly stimulate the growth of probiotics. It was assumed that this was because the EPS had a more complex structure than inulin and glucose, prompting the probiotic to induce multiple metabolic pathways to breakdown the EPS, thus increasing the time for the probiotic to reach maximum viability (Wang et al., 2015; Chen et al., 2019). The four strains showed a general trend of increasing and then decreasing polysaccharide use and their own growth, presumably because the strains reached a growth threshold or metabolized acid production after using polysaccharides, which increased the pH of the medium and limited the growth of the strains (Mohd Nor et al., 2017). The promotion of probiotic proliferation by EPSs is mainly related to the monosaccharide composition, degree of polymerization, type of glycosidic bond, culture conditions and type of probiotic.

## 4. Conclusion

In this study, EPS samples produced by *L. plantarum* HDC-01 was isolated and purified, and the structural composition and functional groups of the isolated EPS were characterized. The EPS had a Mw of 2.505 × 10^6^ Da, as a glucopyranose composed of *α*-(1 → 6) glycosidic bonds and a small number of *α*-(1 → 3) branching structures, and had a smooth and compact surface. We also evaluated the physicochemical properties of the EPS and confirmed that it exhibited good thermal stability, water holding capacity, rheological properties and milk solidification ability. The isolated EPS showed an excellent proliferative effect on intestinal probiotics, revealing the great advantages of EPSs in food and pharmaceuticals, chemical production and other fields. In addition, EPSs usually have an immunostimulatory effect without causing significant side effects. Therefore, future exploration of the immunomodulatory effects of *L. plantarum* EPSs will expand the application and research of EPSs in the field of health medicine.

## Data availability statement

The original contributions presented in the study are included in the article/supplementary material, further inquiries can be directed to the corresponding authors.

## Author contributions

LY: methodology, data curation, and writing—original draft. GY, YZ, and WP: supervision. XQ, BZ, and RD: review and editing. YY and JG: resources and supervision. All authors contributed to the article and approved the submitted version.

## Funding

This work was supported by Outstanding Youth Fund Project of Natural Science Foundation of Heilongjiang Province [no. YQ2021C030] (RD). Project funded by China Postdoctoral Science Foundation [no. 2022MD713755] (RD). Heilongjiang Province Postdoctoral Funding Project [no. LBH-Z21082] (RD). Open Funding Project of Hebei Provincial Key Laboratory of Agricultural Ecological Security [no. 2023SYSJJ17] (RD). Supported Project for “New Era Longjiang Excellent Master’s and Doctoral Dissertations” [no. LJYXL2022-020] (RD). Heilongjiang Province Provincial Colleges and Universities Basic Scientific Research Business Expenses Scientific Research Projects [no. 2022-KYYWF-1075] (RD) and Promotion Project of Basic Ability for Young and Middle-aged Teachers in Universities of Guangxi [nos. 2022KY0541 and 2022KY0547] (GY).

## Conflict of interest

The authors declare that the research was conducted in the absence of any commercial or financial relationships that could be construed as a potential conflict of interest.

## Publisher’s note

All claims expressed in this article are solely those of the authors and do not necessarily represent those of their affiliated organizations, or those of the publisher, the editors and the reviewers. Any product that may be evaluated in this article, or claim that may be made by its manufacturer, is not guaranteed or endorsed by the publisher.
